# Association between living in the endemic area and level of knowledge of visceral leishmaniasis

**DOI:** 10.1186/s12889-024-17775-9

**Published:** 2024-01-24

**Authors:** Eslam Moradi-Asl, Abbas Abbasi-Ghahramanloo, Davoud Adham, Abedin Saghafipour, Kourosh Arzamani, Aboozar Soltani, Jalil Nejati, Samira Firozian, Ali Jalilian, Samad Kazempoor, Mohammad Darvishi, Gorban Mohamad Ozbaki

**Affiliations:** 1 Arthropod-Borne Diseases Research Center, Ardabil University of Medical Sciences, Ardabil, Iran; 2 Department of Public Health, School of Health, Ardabil University of Medical Sciences, Ardabil, Iran; 3 Department of Public Health, School of Health, Qom University of Medical Sciences, Qom, Iran; 4 Vector-borne Diseases Research Center, North Khorasan University of Medical Sciences, North Khorasan, Iran; 5 Research Center for Health Sciences, Institute of Health, Department of Medical Entomology, School of Health, Shiraz University of Medical Sciences, Fars, Iran; 6 Health Promotion Research Center, Zahedan University of Medical Sciences, Zahedan, Iran; 7 Urmia Health Center, Disease Control Unit, Urmia University of Medical Sciences, Urmia, Iran; 8 Department of Public Health, School of Health, Ilam University of Medical Sciences, Ilam, Iran; 9 Kaleybar Health Center, Disease Control Unit, Tabriz University of Medical Sciences, Tabriz, Iran; 10 Health Center of Tangestan, Bushehr University of Medical Sciences, Bushehr, Iran; 11 Gonbad Health Center, Disease Control Unit, Golestan University of Medical Sciences, Golestan, Iran

**Keywords:** Visceral leishmaniasis, Knowledge, Endemic, Iran

## Abstract

**Background:**

Iran is a country with a high prevalence of visceral leishmaniasis (VL) and seven endemic provinces. In this study, we tried to identify unobserved classes of knowledge among Iranians toward VL and assess the predictors of each latent class.

**Methods:**

This cross-sectional study was conducted among randomly selected participants from endemic and non-endemic areas of VL in Iran in 2020 and 2021. The collected data included demographic characteristics and questions about knowledge, attitude, and practice toward VL. We performed latent class analysis using a procedure for latent class analysis (PROC LCA) in SAS to identify the class membership of knowledge of participants toward VL.

**Results:**

Five latent classes were identified: very low (38.9%), low (15.5%), moderate (6.2%), high (14.1%), and very high (25.2%) knowledge about VL. Living in endemic areas significantly increased the odds of belonging to the low (adjusted OR (AOR = 7.23; 95% confidence interval (CI):4.52–11.58), high (AOR = 2.71; 95%CI: 1.73–4.23), and very high (AOR = 8.47; 95%CI: 5.78–12.41) classes compared to the very low class. Also, having academic education increased the odds of membership in the very high class (AOR = 2.36; 95%CI: 1.61–3.47) compared to the very low class.

**Conclusion:**

This study revealed that more than 50% of the participants fell into the latent classes of very low and low knowledge toward VL. Some educational workshops in the endemic areas could be effective in enhancing knowledge about VL.

## Background

Visceral leishmaniasis (VL), the most serious form of leishmaniasis, is caused by *Leishmania* parasites and is characterized as an infectious, systemic, zoonotic, vector-borne, and neglected tropical disease [1, 2]. VL is transmitted by species of Phlebotomus and Lutzomyia sand flies in the old and new worlds, and its reservoirs include humans, dogs, and rodents [3–5]. Most cases occur in Brazil, East Africa, and India. It is estimated that 50 to 90,000 new cases of VL occur in the world annually. However, only 25 to 45% of them have been reported to the World Health Organization (WHO) [3, 4]. One of the countries with a high prevalence of VL is Iran, which has seven endemic regions with 100 to 300 new cases per year, according to reports. Moreover, the disease is reported as sporadic in the rest of Iran [5]. Due to the lack of detection and untimely treatment of VL, 95–100% of the cases could lead to death. Therefore, people’s knowledge, especially in endemic areas, is one of the important strategies for controlling, preventing, and reducing the mortality of VL [6]. Knowledge about the symptoms of the disease, mode of transmission, reservoirs, vectors, controlling and prevention methods, and treatment of the VL can lead to ease of control of the VL in endemic and non-endemic areas. In highly prevalent regions, health education should be prepared in accordance with the structure and population composition [7, 8]. In some executive programs, it has been reported that with coherent educational programs, VL could be controlled [9]. In the implementation of educational programs in endemic areas, the participation of residents is very necessary. So, knowledge of the community about VL is among the most important determinants of community participation [10, 11]. In Iran, there are six endemic areas of VL. Therefore, it is expected that the knowledge of residents of endemic areas about VL is higher than that of people living in non-endemic areas. However, there are no national estimates about the knowledge level of people living in endemic and non-endemic areas. In this study, using the latent class analysis (LCA) model and a cluster analysis model, we sought to identify unobserved classes of knowledge of Iranian people toward VL and assess the predictors of each latent class.

## Methods

### Study area and population

According to the report of the Ministry of Health, in Iran, 7 provinces have been identified as endemic areas of VL. We selected six provinces among them. These six provinces are East Azerbaijan, Ardabil, Fars, North Khorasan, Qom, and Bushehr. Also, to select people from non-endemic regions, we considered geographical and climatic diversity, and four provinces from the west, east, southwest and northwest of the country were selected. These four provinces were Golestan, Sistan and Baluchistan, Ilam and West Azerbaijan. As a result, a 1,000-person sample size from 10 provinces was considered in this study. Five randomly chosen villages from each province, located in various geographic regions, were chosen, and each village responded to 20 questionnaires.

The present cross-sectional study was conducted from October 2020 to February 2021. The selection of participants was done by simple randomization in each village.

### Data collection

All people over 15 years of age could participate in this study, and the inclusion criteria were to be over 15 years old and to have lived in the selected village for more than one year. For under-18-year-old subjects, informed consent was obtained from a parent and/or legal guardian for study participation. There were no exclusion criteria. The needed information was collected through interviews. Each participant completed four sets of questionnaires and checklists. Age, sex, marital status, and other demographic data were evaluated in the first section. The information about participants’ knowledge, attitudes, and practices related to VL was evaluated in the second, third, and fourth parts, respectively. To gather data about the knowledge, attitude, and practice of participants, a standard questionnaire was used [12, 13]. The 10 items of the standard questionnaire were used to subgroup the participants based on their knowledge of VL. These questions collected information on hearing about VL, knowing about the infectiousness of VL, the mode of transmission of VL, the signs and symptoms of VL, the preventability of VL, the cause of VL, the transmission of VL from animals to humans, the outcome of untreated VL, the treatment preference of VL, and the care of a diseased person. In this study, two sections of the standard questionnaire were used: one section focused on 10 questions about knowledge about VL, and the other section focused on demographic characteristics. It should be noted that Cronbach’s Alpha coefficient of the knowledge subscale toward VL was 0.80.

### Data analysis

To identify the best model that could fit the data, we ran LCA, starting with a one-class solution and continuing until there were eight classes. Each LCA model was fit to the data 20 times using different random starting values to investigate model identification. For the selection of the best model, some statistical indices were calculated and compared across eight models, including the likelihood ratio statistics G2, the Akaike information criterion (AIC), the Bayesian information criterion (BIC), entropy, and the log-likelihood value. Among these indices, lower values of G2, AIC, BIC, and the log-likelihood and higher values of entropy indicate a more optimal model fit [14]. Furthermore, in the model selection stage, we have considered the interpretability and parsimony of a model.

Responses to the knowledge questions were categorized as “yes” for having information about each item and “no” for not having it.

A multinomial regression model was performed to examine the association between opted class membership and covariates, with the 1st class being a reference group. The hosmer-Lemeshow guideline was used for variable selection in a multiple-model. Assessed covariates were age, sex (male/female), marital status (single/married), education (lower than high school vs. high school and upper), and living area (endemic vs. non-endemic).

All analyses were performed using SPSS 16 and SAS 9.2 software. Data analysis was done using the chi-square and logistic regression models. To conduct LCA, the PROC LCA was used in SAS 9.2 software. A *P*-value of 0.05 was considered statistically significant.

## Results

**Fig. 1:**
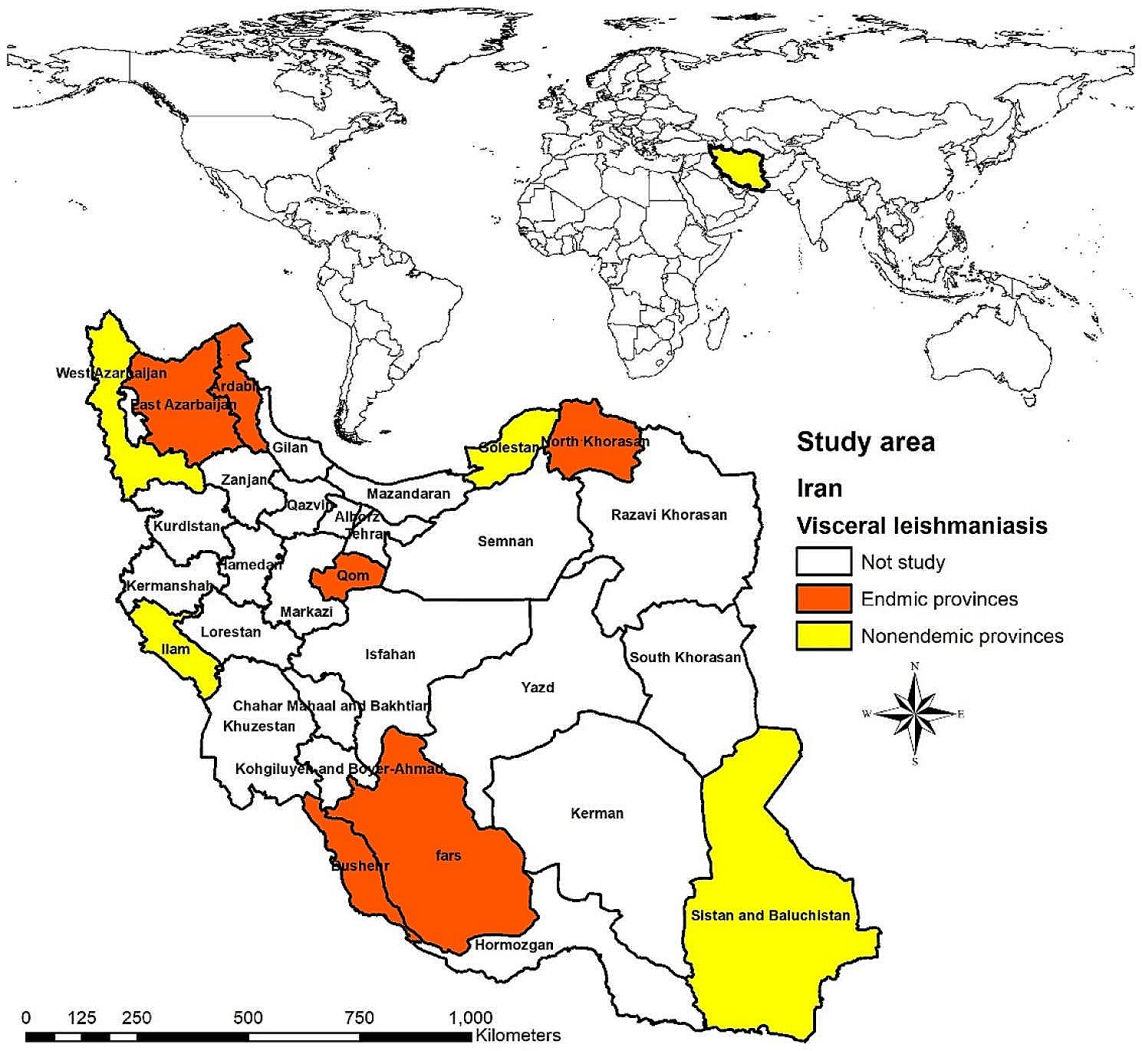
The studied areas include two parts, 6 provinces are endemic areas (red color), 4 provinces are non-endemic areas of visceral leishmaniasis (yellow color), and the rest of the provinces have not been studied (white color)

A total of 934 (response rate = 93.4%) questionnaires were completed and analyzed from 10 provinces (Fig. 1). The mean ± standard deviation (SD) age of participants was 38.29 ± 12.76 years, ranging from 15 to 83 years. Among all participants, 471 (50.4%) were male, and only 158 (16.9%) were single. The educational characteristics revealed that 209 (22.4%) of them had a high school education. Also, 446 (47.8%) of the respondents lived in an endemic area of VL.

A summary of responses to knowledge questions is shown in Table 1. The results suggested that there was good knowledge for some items (i.e., treatment preference and preventability of disease). Also, there was a low level of knowledge about the infectiousness of disease. Table 1 also presents the conditional distribution of living area at each level of the knowledge questions. All items of knowledge about VL have a significant relationship with the living area.

**Table 1:** Percentages of participants responding to questions toward knowledge about Visceral Leishmaniasis by living area in northwest of Iran 2020 and 2021

Items	Living area		*P*-value	Total(*n* = 934)
	Non- Endemic (*n* = 466)	endemic (*n* = 488)		
	n(%)	n(%)		n(%)
**Hearing about VL** ^*^				
no	366(75.2)	214(48.0)	< 0.001	580(62.2)
yes	121(24.8)	232(52.0)		353(37.8)
**Knowing about the infectiousness of the VL** ^*^				
no	384(79.2)	250(56.1)	< 0.001	634(68.1)
yes	101(20.8)	196(43.9)		297(13.9)
**Knowing about the mode of transmission of VL** ^*^				
no	339(70.2)	245(55.3)	< 0.001	584(63.1)
yes	144(29.8)	198(44.7)		342(36.9)
**Knowing about the sign and symptoms of VL** ^*^				
no	338(70.4)	199(45.5)	< 0.001	537(58.6)
yes	142(29.6)	238(54.5)		380(41.4)
**Knowing about the preventability of VL** ^*^				
no	153(36.0)	49(11.6)	< 0.001	
yes	272(64.0)	372(88.4)		644(69.0)
**Knowing about the cause of VL** ^*^				
no	401(83.5)	298(67.4)	< 0.001	699(75.8)
yes	79(16.5)	144(32.6)		223(24.2)
**Knowing about the transmission of VL from animals to humans** ^*^				
no	394(82.1)	257(58.1)	< 0.001	651(70.6)
yes	86(17.9)	185(41.9)		271(29.4)
**Knowing about the outcome of untreated VL** ^*^				
no	386(79.8)	182(41.2)	< 0.001	568(61.3)
yes	98(20.2)	260(58.8)		358(38.7)
**Knowing about the treatment preference of VL** ^*^				
no	65(13.8)	21(4.9)	< 0.001	86(9.5)
yes	406(86.2)	409(95.1)		815(90.5)
**Knowing about the care of diseased person** ^*^				
No	288(59.4)	313(70.8)	< 0.001	601(64.8)
yes	197(40.6)	129(29.2)		326(35.2)

*There were some missing values in some variables

According to the model selection criteria (Table 2), parsimonious, and interpretability of the results, we concluded that the five-class model was appropriate for subgrouping participants. Table 3 presents the results of the LCA model. As mentioned in the Table 3, the first class (very low) described 38.9% of the participants. Also, the second class (low), third class (moderate), fourth class (high), and fifth class (very high) represented 15.5%, 6.2%, 14.1%, and 25.2% of the participants, respectively.

**Table 2:** Comparison of LCA Models With Different Latent Classes Based on Model Selection Statistics

Number of latent class	Number of parameters estimated	G^2^	df	AIC	BIC	Entropy	Maximum log-likelihood
1	10	3069.11	1013	3089.11	3137.50	--	-5469.93
2	21	956.31	1002	998.31	1099.94	0.91	-4413.53
3	32	824.81	991	888.81	1043.67	0.79	-4347.78
4	43	716.51	980	802.51	1010.61	0.81	-4293.63
5	54	635.63	969	743.63	1004.97	0.81	-4253.19
6	65	573.58	958	703.58	1018.15	0.83	-4222.17
7	76	536.76	947	688.76	1056.56	0.83	-4203.76
8	87	500.89	936	674.89	1095.93	0.84	-4185.82

Note. LCA = latent class analysis; AIC = Akaike information criterion; BIC = Bayesian information criterion

**Table 3:** The five Latent Classes Model of knowledge about Visceral Leishmaniasis in Iranian population 2020 and 2021

	Latent class				
	**Very low**	**Low**	**Moderate**	**high**	**Very high**
**Latent class prevalence**	0.389	0.155	0.062	0.141	0.252
**Item-response probabilities**					
**Hearing about VL** ^*^	0.039	0.005	0.005	**0.966**	**0.900**
**Knowing about the infectiousness of the VL**	0.001	0.232	0.005	**0.699**	**0.731**
**Knowing about the mode of transmission of VL**	0.032	0.139	**0.873**	**0.510**	**0.843**
**Knowing about the sign and symptoms of VL**	0.059	0.294	0.141	**0.603**	**0.992**
**Knowing about the preventability of VL**	0.444	**0.912**	**0.866**	**0.862**	**0.988**
**Knowing about the cause of VL**	0.001	0.097	0.117	0.286	**0.709**
**Knowing about the transmission of VL from animals to humans**	0.001	0.445	0.027	0.203	**0.771**
**Knowing about the outcome of untreated VL**	0.096	**0.527**	0.140	0.403	**0.798**
**Knowing about the treatment preference of VL**	**0.812**	**0.856**	**1.00**	**0.982**	**1.00**
**Knowing about the care of a diseased person**	0.251	0.347	**0.994**	0.324	0.364

*Probability of a “Yes” response

Note: The probability of a “No” response can be calculated by subtracting the item-response probabilities shown above from 1

Latent class 1, very low, was characterized by a low probability of a “yes” response to all of the knowledge questions (except treatment preference), and latent class 5, very high, was characterized by a high probability of a “yes” response to all of the questions (except the care of a diseased person). Three other latent classes reflected different patterns of knowledge about VL. Latent class 2, low, was characterized by the high probability of a “yes” response to the three questions of knowledge. Latent class 3, moderate, was characterized by the high probability of a “yes” response to the four questions of knowledge. Finally, latent class 4, high, was characterized by the high probability of a “yes” response to the six questions of knowledge about VL. After identifying the optimal model (the five-class model in this study), we conducted an LCA with covariates to detect the effect of predictors of latent class membership. Table 4 shows the odds ratio of membership in each class compared to the first class associated with living area and other covariates. This index compares the odds of membership in each class with the reference class (i.e. very low group). As shown in Table 4, the odds of membership in classes 3 (OR = 1.09, 95%CI: 1.06–1.12) and 5 (OR = 0.98, 95%CI: 0.96–0.99) associated with age. This table also shows that having a high school education (OR = 2.36, 95%CI: 1.61–3.47) significantly increased the odds of being in class 5. Finally, living in an endemic area significantly increased the odds of being in latent classes 2 (OR = 7.23, 95%CI: 4.52–11.58), 4 (OR = 2.71, 95%CI: 1.73–4.23), and 5 (OR = 8.47, 95%CI: 5.78–12.41). However, living in these areas decreased the odds of membership in class 3 (OR = 0.09, 95%CI: 0.03–0.30). Moreover, Table 4 indicates that the sex of participants and marital status did not have a significant effect on their membership in different classes.

**Table 4:** Predictors of membership in latent classes of knowledge about Visceral Leishmaniasis in Iranian population 2020 and 2021

Predictors	Low	Moderate	high	Very high	*P*-value
	OR(95%CI)	OR(95%CI)	OR(95%CI)	OR(95%CI)	
Age	1.01(0.99–1.03)	1.09(1.06–1.12)	0.99(0.97–1.01)	0.98(0.96–0.99)	< 0.001
Being male	0.76(0.50–1.16)	2.70(1.39–5.25)	0.98(0.66–1.44)	0.93(0.66–1.31)	0.2954
Bing single	0.72(0.39–1.38)	3.68(1.75–7.72)	1.12(0.68–1.84)	0.75(0.45–1.17)	0.2576
Education (academic)	0.91(0.53–1.57)	0.54(0.23–1.30)	1.20(0.76–1.91)	2.36(1.61–3.47)	0.0003
Living in endemic area	7.23(4.52–11.58)	0.09(0.03–0.30)	2.71(1.73–4.23)	8.47(5.78–12.41)	< 0.001

The reference class: C1

OR: odds ratio

CI: confidence interval

## Discussion

This study evaluated the pattern of knowledge toward VL among Iranian people with the LCA approach. We were able to find five distinct classes of knowledge named very low, low, moderate, high, and very high that represent 38.9%, 15.5%, 6.2%, 14.1%, and 25.2% of the participants in our sample.

The results indicated that there is a significant relationship between knowledge indicators and living areas. The present study also revealed that living in endemic areas vs. non-endemic ones significantly increased the odds of membership in the second (OR = 7.23, 95%CI: 4.52–11.58), fourth (OR = 2.71, 95%CI: 1.73–4.23), and fifth (OR = 8.47, 95%CI: 5.78–14.41) classes in comparison to the first class. This might be because most people who live in endemic regions frequently interact with VL-related information, which means they may know more about the disease than those who reside in non-endemic regions. However, in areas where VL is endemic, patients and family members have received the necessary education and training from healthcare professionals. They might consequently serve as effective health community agents. Due to the different endemic areas and to avert the spreading of the disease to areas that are non-endemic for VL in Iran, for increasing awareness activities, the involvement of health workers and the school in the community is needed on a large scale.

To the best of our knowledge, this study is the first attempt to use LCA for the subgrouping of participants based on knowledge levels about VL. However, some studies have assessed knowledge of VL in different strata of the population with other approaches. Gelaye et al. reported that 33.2% of migrants and seasonal farmworkers in Ethiopia have good knowledge of VL. In this study, the knowledge of participants was considered low-level [15]. Studies from Addis Zemen, Ethiopia [12], Western Tigray, Ethiopia [10], and India [16] indicated that 89.4%, 59%, and 43.9% of the participants have good knowledge of VL, respectively. In our study, more than half of the participants had very low or low knowledge about VL and only 25.2% of them had very high knowledge. Differences in the study population could affect the levels of knowledge in different studies. For example, a study among HIV-positive people showed that these participants had insufficient knowledge about VL transmission, vector breeding sites, time or season of bite, and prevention strategies for VL [17].

In addition to the study population, the variability between studies might be due to the lack of community health education, community awareness, and socioeconomic status of the studied areas [12]. Inconsistent with other studies [10, 12, 18, 19], our study revealed that after adjusting for other factors, having an academic educational level increased the odds of belonging to the fifth class (i.e., very high) compared to the first class (very low). So, considering educational campaigns and persistent activities toward behavioral change could be an important factor in increasing knowledge about VL.

The present study indicated that age is significantly associated with latent classes of knowledge toward VL. With the increasing age of the participants, the odds of being in latent class 3 (moderate) (OR = 1.09, 95%CI: 1.06–1.12) increased in comparison to latent class 1 (very low). On the other hand, the odds of membership in class 5 (very high) (OR = 0.98, 95%CI: 0.96–0.99) decreased compared to class 1 (very low). Studies from Ethiopia [15] and Paraguay [20] revealed that age significantly affects the odds of having good knowledge about VL and with increasing the age of the participants, the odds of having good knowledge increased significantly. Decreasing the odds of membership in the fifth class in our study requires more investigations in different groups and areas.

## Conclusion

This study revealed that 39.3% of the participants belonged to very high or high classes. In other words, less than half of the participants had acceptable knowledge about VL. However, a large percent of the participants fell into the latent classes of very low and low knowledge toward VL. This study showed that among participants with very high knowledge, most of them didn’t know about the care of a diseased person. We found that education and living in endemic areas were associated with having very high knowledge about VL. Consequently, focusing on the knowledge of the people toward VL may be helpful in designing and executing effective programs to reduce VL prevalence in Iran. Also, some educational workshops in the endemic areas could be effective in enhancing knowledge about VL.

## Data Availability

The data used in the observational study are available from the corresponding author upon reasonable request.
